# Identification of thresholds for accuracy comparisons of heart rate and respiratory rate in neonates

**DOI:** 10.12688/gatesopenres.13237.2

**Published:** 2021-10-08

**Authors:** Jesse Coleman, Amy Sarah Ginsburg, William M. Macharia, Roseline Ochieng, Guohai Zhou, Dustin Dunsmuir, Walter Karlen, J. Mark Ansermino

**Affiliations:** 1 Evaluation of Technologies for Neonates in Africa (ETNA), Aga Khan University Hospital, Nairobi, Kenya; 2 University of Washington, Seattle, WA, 98195, USA; 3 Department of Paediatrics, Aga Khan University Hospital, Nairobi, Kenya; 4 Center for Clinical Investigation, Brigham and Women's Hospital, Boston, MA, 02115, USA; 5 Anesthesiology, Pharmacology & Therapeutics, The University of British Columbia, Vancouver, BC, V6T 1Z3, Canada; 6 Mobile Health Systems Lab, Department of Health Sciences and Technology, ETH Zürich, Zürich, 8092, Switzerland

**Keywords:** neonatal vital sign measurement, monitoring, heart rate, respiratory rate, accuracy, validation

## Abstract

**Background: **Heart rate (HR) and respiratory rate (RR) can be challenging to measure accurately and reliably in neonates. The introduction of innovative, non-invasive measurement technologies suitable for resource-constrained settings is limited by the lack of appropriate clinical thresholds for accuracy comparison studies.

**Methods: **We collected measurements of photoplethysmography-recorded HR and capnography-recorded exhaled carbon dioxide across multiple 60-second epochs (observations) in enrolled neonates admitted to the neonatal care unit at Aga Khan University Hospital in Nairobi, Kenya. Trained study nurses manually recorded HR, and the study team manually counted individual breaths from capnograms. For comparison, HR and RR also were measured using an automated signal detection algorithm. Clinical measurements were analyzed for repeatability.

**Results: **A total of 297 epochs across 35 neonates were recorded. Manual HR showed a bias of -2.4 (-1.8%) and a spread between the 95% limits of agreement (LOA) of 40.3 (29.6%) compared to the algorithm-derived median HR. Manual RR showed a bias of -3.2 (-6.6%) and a spread between the 95% LOA of 17.9 (37.3%) compared to the algorithm-derived median RR, and a bias of -0.5 (1.1%) and a spread between the 95% LOA of 4.4 (9.1%) compared to the algorithm-derived RR count. Manual HR and RR showed repeatability of 0.6 (interquartile range (IQR) 0.5-0.7), and 0.7 (IQR 0.5-0.8), respectively.

**Conclusions: **Appropriate clinical thresholds should be selected *a priori* when performing accuracy comparisons for HR and RR. Automated measurement technologies typically use a smoothing or averaging filter, which significantly impacts accuracy. A wider spread between the LOA, as much as 30%, should be considered to account for the observed physiological nuances and within- and between-neonate variability and different averaging methods. Wider adoption of thresholds by data standards organizations and technology developers and manufacturers will increase the robustness of clinical comparison studies.

## Introduction

There is a high risk of mortality during the neonatal period, particularly in resource-constrained settings ^1^. Continuous monitoring of neonatal vital signs enables early detection of physiological deterioration and potential opportunities for lifesaving interventions ^2– 4^. The development of new, innovative, non-invasive, multiparameter continuous physiological monitors specifically for neonates offers the promise of improving clinical outcomes in this vulnerable population. However, before use, these technologies should be tested in real-world situations to determine accuracy and clinical feasibility.

A neonate's marked physiological variability, small size, and often fragile condition can offer challenges when measuring and monitoring vital signs. A lack of neonatal clinical validation standards further undermines the development of continuous monitors clinically validated specifically for neonates. Determining the accuracy of new continuous monitors is an essential step in bringing these technologies to market ^5, 6^.

The Evaluation of Technologies for Neonates in Africa (ETNA) platform aims to independently establish the accuracy and feasibility of novel continuous monitors suitable for use in neonates in resource-constrained settings ^7^. To determine accuracy and agreement, new technologies are compared against existing reference methods or technologies ^8^. Before the comparison process can proceed, a clinical reference verification step is necessary to determine appropriate accuracy thresholds ^7^. These *a priori* thresholds determine the target level of agreement required and thus, the success or failure of an investigational technology. This study describes the verification processes we conducted with a clinical reference technology in order to determine appropriate heart rate (HR) and respiratory rate (RR) accuracy thresholds to use in subsequent new continuous monitors accuracy comparisons.

## Methods

### Study design

This was a cross-sectional study which aimed to identify the natural variation in neonatal HR and RR in order to identify appropriate accuracy thresholds for use in an accuracy comparison of continuous monitors.

### Setting and participants

Study participants were neonates admitted for observation and care in the maternity ward, neonatal intensive care, and the neonatal high dependency units at Aga Khan University Hospital in Nairobi, Kenya (AKUHN). Between June and August 2019, caregivers were approached, recruited, and sequentially screened for enrolment by trained study staff during routine newborn intake procedures. To minimize potential selection bias, all caregivers were approached in a sequential manner, as much as possible and introduced to the study using a standardized recruitment script. Final eligibility determination was dependent on medical history results, physical examination, an appropriate understanding of the study by the caregiver, and completion of the written informed consent process ( Table 1).

**Table 1.** Study eligibility criteria and definitions.

Eligibility criteria	
Inclusion criteria	• Male or female neonate, corrected age of <28 days • Willingness and ability of neonate’s caregiver to provide informed consent and to be available for follow-up for the planned duration of the study
Exclusion criteria	• Receiving mechanical ventilation or continuous positive airway pressure • Skin abnormalities in the nasopharynx and/or oropharynx • Contraindication to the application of skin sensors • Known arrhythmia • Any medical or psychosocial condition or circumstance that, in the opinion of the investigators, would interfere with the conduct of the study or for which study participation might jeopardize the neonate’s health
Study definitions	
Epoch	A 60-second period of time
Heartbeat	One pulsation of the heart, including one complete contraction and dilatation
Heart rate (HR)	Number of heart beats within an epoch
Breath	One cycle of inhalation and exhalation
Breath duration	Length of time from the start to the end of a single breath
Respiratory rate (RR)	Number of breaths initiated within an epoch
Pulse oximetry signal quality index (PO-SQI)	Automated indicator of signal quality from the plethysmographic recording.
CO _2_-SQI	Algorithm-defined indicator of signal quality from the capnography channel
Accuracy	The closeness a measured value is from the true value
Repeatability	The closeness of the results of successive measurements of the same measure
Agreement (between measures)	The consistency between two sets of measurements
Accuracy Threshold	A pre-specified value used to determine if a set of measurements has achieved a sufficient accuracy when compared with a reference value
Precision	The closeness of measurements to each other

### Study procedures

The Masimo Rad-97 Pulse CO-Oximeter® with NomoLine Capnography (Masimo Corporation, Irvine, CA, USA) was selected as the reference technology based on validated oxygen saturation (SpO _2_) accuracy measurement in neonates ^9– 11^. During study participation, trained and experienced study nurses attached the Rad-97 to neonates and conducted manual HR measurements (counting over 60-second epochs) every 10 minutes for the first hour and once per hour of participation thereafter, following World Health Organization (WHO) guidance for HR measurement in neonates ^12^. Photoplethysmographic HR was also measured via the Masimo Rad-97 pulse oximetry skin sensor attached to the neonate’s foot. RR was measured by capnography using an infant/pediatric nasal cannula to collect the neonate’s exhaled carbon dioxide (CO _2_) levels. Duration of data collection length was set at a minimum of one hour, with no upper limit. Neonates exited from the study upon discharge from the ward or by caregiver request.

### Data collection and analysis

Using a custom Android (Google, Mountain View, CA, USA) application, raw data was collected from the Masimo Rad-97 in real-time through a universal serial bus (USB) asynchronous connection and parsed in C (Dennis Ritchie & Bell Labs, USA). Instantaneous HR was obtained from the timing of the pulse oximetry signal quality index (PO-SQI). The plethysmogram waveform was sampled at 62.5 Hz with the PO-SQI identified by the Masimo Rad-97 at the peak of each heartbeat. The CO _2_ waveform was sampled at approximately 20 Hz from the capnography channel. The parsed output included an accurate time stamp for each entry in the waveform data output to facilitate synchronization and analysis. Data were recorded and stored on a secure AKUHN-hosted REDCap server ^13^.

We analyzed the CO _2_ waveform data using a breath detection algorithm developed in MATLAB (Math Works, USA) and based on adaptive pulse segmentation ^14^. In addition to providing a RR, the algorithm analyzed the waveform’s shape and identified the breath duration (waveform trough to trough) for each breath. From the breath duration, we calculated a RR based on the median breath duration within the epoch. We developed a custom capnography quality score (CO _2_-SQI) based on capnography features to assist with data selection. HR and RR counts and medians, along with signal quality metrics from the MATLAB signal detection algorithm, were analyzed using R version 4.0.3 ^15^. Capnogram waveforms were generated with two seconds added at the beginning and end of each epoch to facilitate manual breath counting within the epoch.

To ensure temporal alignment between measurements, HR and RR epochs were synchronized across source data devices. For HR, alignment was done using a timestamp in REDCap that was set by the study nurse as HR counting was initiated. Before analysis, this timestamp was synchronized with the same timestamp in the custom Android application. Both the REDCap and Android servers were connected via the internet to a Network Time Protocol (NTP) server. Alignment of RR epochs was based on the Android application timestamp. All RR waveforms were compared visually to further ensure epoch synchronization.

One of the authors (JMA, a pediatric anesthesiologist) reviewed the capnogram tracings and discarded plots with marked variability or a significant duration of an artifact that would have made breaths challenging to count. The remaining plots were provided to two trained observers to independently count all breaths within each epoch using a set of predefined rules created by the investigators ( Table 2). The two independent counts were averaged, and if the number of breaths counted by the two observers varied by more than three breaths per epoch, a third trained observer independently counted the plot, and the two closest counts were averaged.

**Table 2.** Rules for identifying breaths based on graphical waveform plots.

1. Count peaks of the waveform that are within the white background. Ignore peaks that are within the grey background on either side of the image.
2. A peak should be counted as a breath when the peak of the waveform is above 15mmHg, the lower horizontal blue line.
3. If the peak does not reach the lower horizontal blue line at 15 mmHg, to be counted as a breath, the peak should reach at least 50% of the mean peak.
4. The waveform should dip down to the normal baseline (either below 15 mmHg, the lower horizontal blue line, or based on other breaths). If the waveform does not reach below this point, then this is considered part of the same (double) peak and only counted as a breath once.

Measurement repeatability was estimated using linear mixed-effects models based on the between- and within-neonate variability for each data source using R version 4.0.3 ^16^. Agreement between data collection methods was assessed using the method described by Bland-Altman for replicated observations and reported as a mean bias with 95% confidence intervals (CIs), 95% upper and lower limits of agreement (LOA), and as a root mean square deviation (RMSD) ^17^. The aim was to identify practical threshold limits using data from the clinical reference technology verification process.

### Sample size

We estimated that 20 neonates with ten replications each would give a 95% CI LOA between two methods of +/-0.76 times the standard deviation (SD) of their differences. Sample size estimates for method comparison studies typically depend on the CI required around the LOA, and sample sizes of 100 to 200 provide tight CIs ^17^. We aimed for a sample size of at least 30 neonates to ensure a diverse population and sufficient replications for tight CIs.

### Ethical approval

The study was conducted per the International Conference on Harmonisation Good Clinical Practice and the Declaration of Helsinki 2008. The protocol and other relevant study documents were approved by Western Institutional Review Board (20191102; Puyallup, Washington, USA), Aga Khan University Nairobi Research Ethics Committee (2019/REC-02 v2; Nairobi, Kenya), Kenyan Pharmacy and Poisons Board (19/05/02/2019(078)) and Kenyan National Commission for Science, Technology and Innovation (NACOSTI/P/19/68024/30253). Written informed consent was obtained in English or Swahili by trained study staff from each neonate’s caregiver according to a checklist that included ascertainment of caregiver comprehension.

## Results

**Figure 1.**
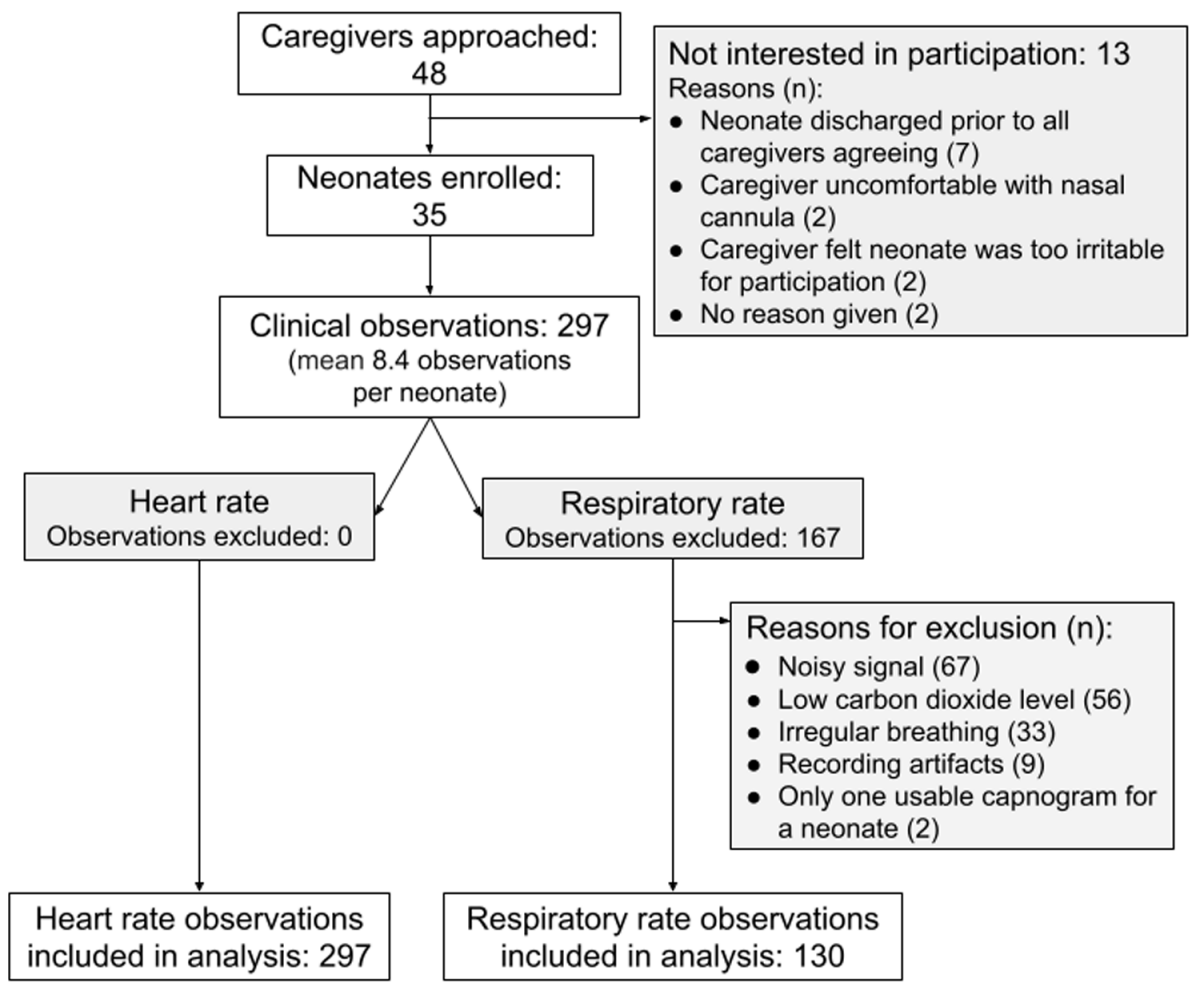
Recruitment flow chart.

Between June and August 2019, 35 neonates were enrolled, and 297 clinical observations were completed with a mean of 8.4 (SD 1.7) observations per neonate ( Table 3; Figure 1) and a median data collection time of 4 hours, 5 minutes (interquartile range (IQR) 3:52-4:45) ^18^. The manual HR measurements were found to have a non-normal distribution with skewness of 0.76 and kurtosis of 3.60 (p<0.001). The median manual HR measurement for all observations was 134 (IQR 126-143) beats per minute (bpm).

**Table 3.** Neonate demographic data.

Sex			Age at participation (days)		Gestation at birth (weeks)		Weight at birth (grams)	
Female	Male	Other	Median	IQR	Median	Range	Median	IQR
22	13	0	2	0-4	33	32-34	1500	1260-1600

**Figure 2.**
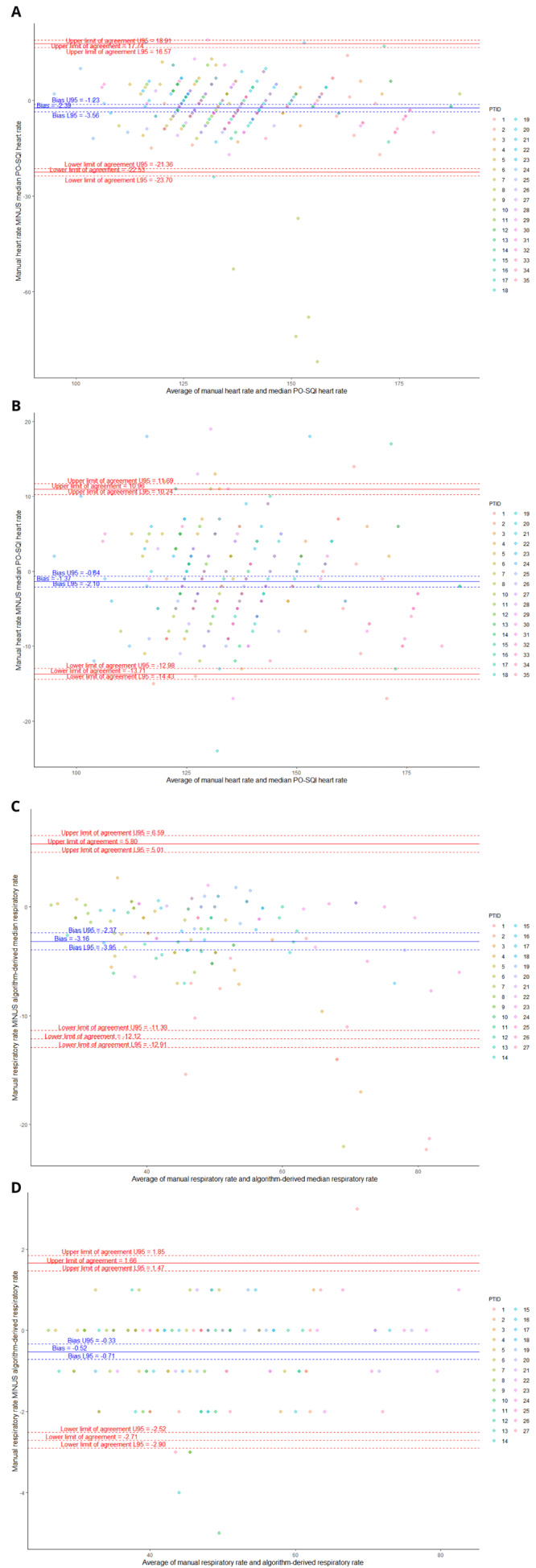
Bland-Altman plots comparing manual heart rate (HR) vs median pulse oximetry signal quality index (PO-SQI) HR for all epochs ( **A**), modified manual HR vs median PO-SQI HR with PTID9 removed due to significant outliers ( **B**), manual respiratory rate (RR) vs algorithm-derived median RR ( **C**), and manual RR vs algorithm-derived RR count ( **D**).

The manual HR demonstrated a negative bias of -2.4 (-1.8%) compared to the median PO-SQI HR, and a marked spread between the 95% LOA of 40.3 (29.6%). The RMSD was 10.5 (7.7%). Removing data from a single outlier neonate resulted in a smaller bias of -1.4 (-1.0%), a tighter spread between the 95% LOA of 24.7 (18.2%), and a lower RMSD of 6.4 (4.7%) ( Table 4; Figure 2).

**Table 4.** Bland-Altman analysis of heart rate (HR) and respiratory rate (RR) methods.

	Bias (normalized)	95% upper/ lower limits of agreement	Spread of 95% limits of agreement (normalized)	Root-mean- square deviation (normalized)
Heart rate				
Manual HR vs median pulse oximetry signal quality index HR	-2.39 (-1.8%)	-22.53/17.74	40.27 (29.6%)	10.5 (7.7%)
Manual HR vs median pulse oximetry signal quality index HR (outlier neonate removed)	-1.4 (-1.0%)	-13.71/10.97	24.67 (18.2%)	6.4 (4.7%)
Respiratory rate				
Manual RR vs algorithm-derived median RR	-3.16 (-6.6%)	-12.1/5.8	17.9 (37.3%)	5.5 (11.4%)
Manual RR vs algorithm-derived RR count	-0.52 (-1.1%)	-2.7/1.66	4.37 (9.1%)	1.2 (2.5%)

**Figure 3.**
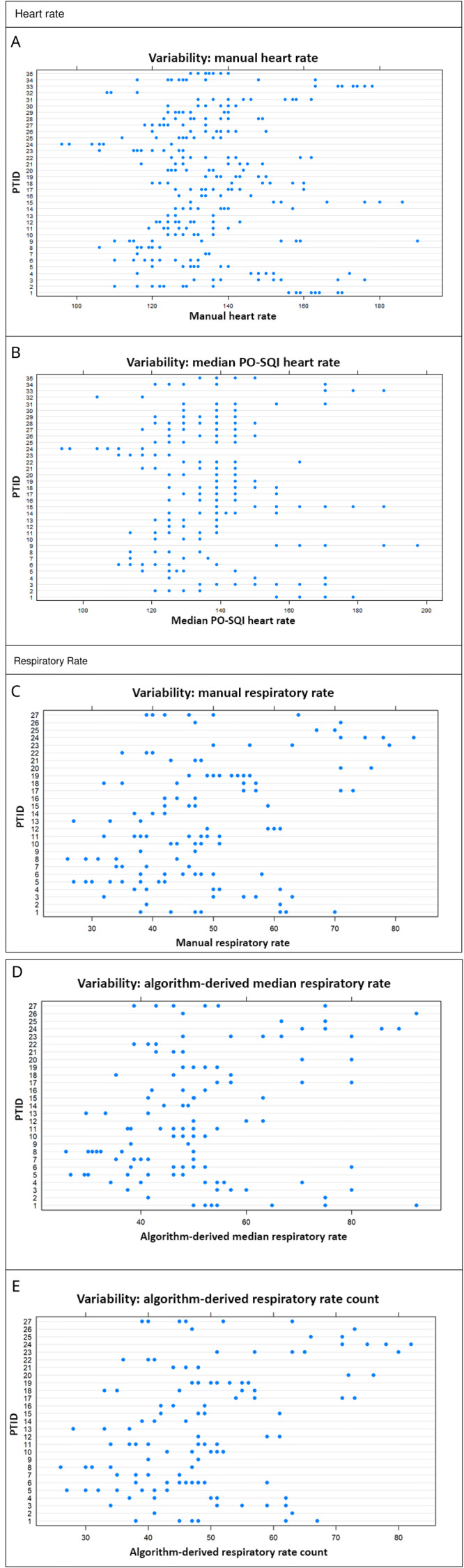
Variability plots (vertical for between-neonate variability, horizontal for within-neonate variability). Manual heart rate (HR) between-neonate variability accounts for 62% of total variability ( **A**); median pulse oximetry signal quality index (PO-SQI) HR between-neonate variability accounts for 75% of total variability ( **B**); manual respiratory rate (RR) between-neonate variability accounts for 66% of total variability ( **C**); algorithm-derived median RR between-neonate variability accounts for 50% of total variability ( **D**); and algorithm-derived RR count between-neonate variability accounts for 66% of total variability ( **E**).

Moderate repeatability was demonstrated with approximately 62% (95% CI 47%-73%) of the manual HR variability being due to differences between neonates ( Table 5, Figure 3A). Since the 95% CI for manual HR crossed 50%, the between- and within-neonate variability appeared to be comparable, with neither causing significantly more variability than the other.

**Table 5.** Repeatability results for heart rate (HR) and respiratory rate (RR) measurements for all included epochs.

	Repeatability ^1^ (95% Confidence Intervals)
Heart rate (n=297 epochs)	
Manual HR	0.62 (0.47-0.73)
Median pulse oximetry signal quality index HR	0.75 (0.62-0.83)
Respiratory rate (n=130 epochs)	
Manual RR	0.66 (0.47-0.79)
Algorithm-derived median RR	0.50 (0.28-0.67)
Algorithm-derived RR count	0.66 (0.46-0.79)

^1^ Repeatability = (between-neonate variance/(between-neonate variance + within-neonate variance))

Manual RR from capnograms were found to have a non-normal distribution with skewness of 0.61 and kurtosis of 2.96 (p=0.027). The median manual RR measurement for all observations was 47 (IQR 39-56) breaths per minute. The manual RR compared to the algorithm-derived median RR showed a negative bias of -3.2 (-6.6%) and a marked spread between the 95% LOA of 17.9 (37.3%). The RMSD was 5.5 (11.4%). Comparing the manual RR to the algorithm-derived RR count showed a smaller bias of -0.5 (-1.1%) and a tighter spread between the 95% LOA of 4.4 (9.1%). The RMSD was 1.2 (2.5%).

The repeatability was moderate with approximately 66% (95 CI 47%-79%) of the manual RR variability due to differences between neonates ( Table 5, Figure 3C). Since the 95% CI crossed 50%, the amount of between- and within-neonate variability appeared similar, with neither one resulting in significantly more variability than the other.

## Discussion

This reference technology clinical verification study showed minimal measurement bias with a wide spread of 95% upper and lower LOAs and similar repeatability compared with manual clinical measurements. The agreement results allowed us to identify practical HR and RR thresholds for our subsequent technology comparison evaluation. Specifically, we identified a 30% spread between the 95% upper and lower LOA. These *a priori*-defined thresholds were based on variability observed ten and sixty minutes apart in the same neonate and considered the natural within-neonate physiologic variability. Variability was found to be more marked in some neonates. In part, the 30% spread between 95% upper and lower LOA was selected based on the idea that thresholds should not be more stringent than the observed physiological variability, and in part, based on results from the different averaging methods (manual RR vs algorithm-derived median RR). Given the large difference in results between the two averaging methods, considerable thought should be given prior to choosing an averaging method. A random selection of real clinical data can provide appropriate guidance for selecting suitable neonatal accuracy thresholds.

Of note, one neonate (PTID9) significantly impacted the LOA for HR. Five of nine of this neonate’s manual HR measurements significantly diverged from the same epoch’s PO-SQI HR values and were significantly lower than their mean PO-SQI HR, despite having acceptable signal quality scores. This irregularity suggests a HR reading or data entry error by the study nurse. Removing this neonate’s data and re-analyzing it resulted in a smaller bias and tighter LOAs ( Figure 2B).

Results from this clinical verification highlight the difficulty with existing performance thresholds. Current United States Food and Drug Administration performance thresholds for HR measurement, based on electrocardiogram measurements, may not be applicable for use in neonates or when using photoplethysmography for estimating HR ^19^. The current UNICEF target product profile for RR measurement technology recommends a ±2 breaths per minute threshold, which may be too stringent even for use in adults ^20, 21^. Using a ±2 breaths per minute recommendation with our RR data would result in a LOA spread threshold of no more than 5%, which is half the LOA spread of our best performing RR comparison. Furthermore, a ±2 breaths per minute or 5% spread in LOA is smaller than random and natural within-neonate physiologic variability (11.5% in this study [unpublished data]) and would result in unrealistically stringent thresholds.

Selecting a performance threshold is challenging. The threshold cannot be too restrictive or inflexible, thereby stifling innovation and preventing new single or multi-parameter continuous monitors from reaching the market. However, too lax a threshold could result in an inaccurate representation of the underlying physiological state. One key limitation is that the true underlying HR or RR is unknown, regardless of the measurement method ^6, 22^. The primary goal of this reference technology verification study was to establish *a priori* thresholds as the first step of our technology comparison evaluation while at the same time understanding that the true underlying RR and HR cannot be known and also recognizing the marked physiologic variability between and within neonates.

In this study, we did not attempt to define or detect clinically meaningful events; instead, we focused on describing non-random thresholds that fall outside of normal physiological variability. We defined HR and RR thresholds based on the difference between the 95% upper and lower LOA. Additional studies will be required to determine if these thresholds translate into improved clinical outcomes.

Performance thresholds identified using this method are influenced by the characteristics of the neonates studied, the data selection methods, and the number of comparisons. For this reason, the thresholds we identified may not be applicable in different neonate cohorts, such as those receiving mechanical ventilation or immediately following birth, among others. Variability will be influenced by disturbances in the environment such as routine procedures, feeding, noise, and time of day. To minimize variability in our data set, we used only RR epochs that appeared to be regular based on visual inspection. Although these segments were selected based on predefined criteria, a majority (167/297) were discarded as the extreme variability seen in some recordings would have made reproducible manual counting of breaths impossible. We have previously demonstrated acceptable agreement between ECG derived HRV and PPG derived HRV in children with an appropriate sampling rate of the PPG. This should be validated in neonates using an ECG ^23^.

## Conclusion

Appropriate clinical thresholds should be selected *a priori* when performing accuracy comparisons for HR and RR. The magnitude and importance of sample size, as well as within-neonate variability requires further investigation. A larger sample size could allow the development of an error model that more clearly describes the error due to various factors such as the measurement technology, averaging method, the observer, and the natural variability of neonatal HR and RR. We strongly support the creation of international standards for technology comparison studies in neonates. These standards should include thresholds for HR and RR based on the specific neonatal population studied and provide details of the experimental conditions, data selection methods, and analysis methods used. Together, such standards would lay the groundwork for a robust continuous monitor comparison field.

## Data availability

### Underlying data

Dryad: Identification of thresholds for accuracy comparisons of heart rate and respiratory rate in neonates. https://doi.org/10.5061/dryad.1c59zw3vb ^18^.

This project contains the following underlying data:

- Coleman-2021-ETNA-DemographicData.csv
- Raw data folder (contains all raw capnography and pleth data)
- Coleman-2021-ETNA-ProcessedPulseValues.csv
- Coleman-2021-ETNA-ProcessedRespirationValues.csv

Data are available under the terms of the Creative Commons Zero "No rights reserved" data waiver (CC0 1.0 Public domain dedication).

## References

[1] United Nations Inter-agency Group for Child Mortality Estimation (UN IGME): Levels & Trends in Child Mortality: Report 2020, Estimates developed by the UN Inter-agency Group for Child Mortality Estimation.New York: United Nations Children’s Fund,2020. Reference Source

[2] Fairchild KD Schelonka RL Kaufman DA : Septicemia mortality reduction in neonates in a heart rate characteristics monitoring trial. *Pediatr Res.* 2013;74(5):570–5. 10.1038/pr.2013.136 23942558 PMC4026205

[3] Warburton A Monga R Sampath V : Continuous pulse oximetry and respiratory rate trends predict short-term respiratory and growth outcomes in premature infants. *Pediatr Res.* 2019;85(4):494–501. 10.1038/s41390-018-0269-4 30679791

[4] Kumar N Akangire G Sullivan B : Continuous vital sign analysis for predicting and preventing neonatal diseases in the twenty-first century: big data to the forefront. *Pediatr Res.* 2020;87(2):210–20. 10.1038/s41390-019-0527-0 31377752 PMC6962536

[5] Harris BU Char DS Feinstein JA : Accuracy of Pulse Oximeters Intended for Hypoxemic Pediatric Patients. *Pediatr Crit Care Med.* 2016;17(4):315–20. 10.1097/PCC.0000000000000660 26914626

[6] Ansermino JM Dumont G Ginsburg AS : How Uncertain Is Our Reference Standard for Respiratory Rate Measurement? *Am J Respir Crit Care Med.* 2019;199(8):1036–7. 10.1164/rccm.201812-2266LE 30673505

[7] Ginsburg AS Nkwopara E Macharia W : Evaluation of non-invasive continuous physiological monitoring devices for neonates in Nairobi, Kenya: a research protocol. *BMJ Open.* 2020;10(4):e035184. 10.1136/bmjopen-2019-035184 32284391 PMC7200030

[8] Goldsack JC Coravos A Bakker JP : Verification, analytical validation, and clinical validation (V3): the foundation of determining fit-for-purpose for Biometric Monitoring Technologies (BioMeTs). *NPJ Digit Med.* 2020;3:55. 10.1038/s41746-020-0260-4 32337371 PMC7156507

[9] Lee HJ Choi JH Min SJ : Comparison of the Clinical Performance between Two Pulse Oximeters in NICU: Nellcor N-595(R) versus Masimo SET(R). *Journal of the Korean Society of Neonatology.* 2010;17(2):245–249. 10.5385/jksn.2010.17.2.245

[10] Singh JKSB Kamlin COF Morley CJ : Accuracy of pulse oximetry in assessing heart rate of infants in the neonatal intensive care unit. *J Paediatr Child Health.* 2008;44(5):273–5. 10.1111/j.1440-1754.2007.01250.x 17999668

[11] Hay WW Jr Rodden DJ Collins SM : Reliability of conventional and new pulse oximetry in neonatal patients. *J Perinatol.* 2002;22(5):360–6. 10.1038/sj.jp.7210740 12082469

[12] World Health Organization: Integrated management of childhood illness: caring for newborns and children in the community.2011. Reference Source

[13] Harris PA Taylor R Thielke R : Research electronic data capture (REDCap)--a metadata-driven methodology and workflow process for providing translational research informatics support. *J Biomed Inform.* 2009;42(2):377–81. 10.1016/j.jbi.2008.08.010 18929686 PMC2700030

[14] Karlen W Ansermino JM Dumont G : Adaptive pulse segmentation and artifact detection in photoplethysmography for mobile applications. *Annu Int Conf IEEE Eng Med Biol Soc.* 2012;2012:3131–4. 10.1109/EMBC.2012.6346628 23366589

[15] R Core Team: R: A language and environment for statistical computing. Reference Source

[16] Stoffel MA Nakagawa S Schielzeth H : rptR: repeatability estimation and variance decomposition by generalized linear mixed‐effects models. *Methods Ecol Evol.* 2017;8(11):1639–44. 10.1111/2041-210X.12797

[17] Bland JM Altman DG : Measuring agreement in method comparison studies. *Stat Methods Med Res.* 1999;8(2):135–60. 10.1177/096228029900800204 10501650

[18] Coleman J : Identification of thresholds for accuracy comparisons of heart rate and respiratory rate in neonates.Dryad, Dataset,2021. 10.5061/dryad.1c59zw3vb PMC8630397 34901754

[19] American National Standards Institute, Inc: Cardiac monitors, heart rate meters, and alarms.{Association for the Advancement of Medical Instrumentation}2002.

[20] UNICEF: Target Product Profile - Respiratory Rate Monitor / Apnea Monitor.2020. Reference Source

[21] Ermer S Brewer L Orr J : Comparison of 7 Different Sensors for Detecting Low Respiratory Rates Using a Single Breath Detection Algorithm in Nonintubated, Sedated Volunteers. *Anesth Analg.* 2019;129(2):399–408. 10.1213/ANE.0000000000003793 30234539

[22] Ginsburg AS Lenahan JL Izadnegahdar R : A Systematic Review of Tools to Measure Respiratory Rate in Order to Identify Childhood Pneumonia. *Am J Respir Crit Care Med.* 2018;197(9):1116–27. 10.1164/rccm.201711-2233CI 29474107

[23] Dehkordi P Garde A Karlen W : Pulse rate variability compared with Heart Rate Variability in children with and without sleep disordered breathing. *Annu Int Conf IEEE Eng Med Biol Soc.* 2013;2013:6563–6566. 10.1109/EMBC.2013.6611059 24111246

