# Supplementary material for: Identification of thresholds for accuracy comparisons of heart rate and respiratory rate in neonates
Source: Gates Open Res. 2021 Oct 8;5:93. Originally published 2021 Jun 10. [Version 2] doi: 10.12688/gatesopenres.13237.2 (PMC8630397; doi:10.12688/gatesopenres.13237.2)
Supplement: Peer review history

# Peer review: Reviewer response for version 2

**DOI:** 10.21956/gatesopenres.14653.r31258

**Published:** 2021-11-29

**Authors:** AbdelKebir Sabil

**Affiliations:** 1 Cloud Sleep Lab, Paris, France

The method and the results of this study are of interest and the authors seem to have answered the concerns expressed by the reviewers after the initial submission. Despite the fact that I did not participate in the first review of the manuscript, I believe that it would have been interesting if data comparing heart rate variability measured using ECG to pulse rate variability measured with the oximeter. In that case, indices in the time domain (SDNN and RMSSD) and frequency domain (LF, HF and LF/HF) should be compared using different methods. The other comment I have is that the sample size is a bit too small.

Is the work clearly and accurately presented and does it cite the current literature?

Yes

If applicable, is the statistical analysis and its interpretation appropriate?

Yes

Are all the source data underlying the results available to ensure full reproducibility?

Yes

Is the study design appropriate and is the work technically sound?

Partly

Are the conclusions drawn adequately supported by the results?

Yes

Are sufficient details of methods and analysis provided to allow replication by others?

Yes

Reviewer Expertise:

Technology clinical validation expert.

I confirm that I have read this submission and believe that I have an appropriate level of expertise to confirm that it is of an acceptable scientific standard.

# Peer review: Reviewer response for version 2

**DOI:** 10.21956/gatesopenres.14653.r31254

**Published:** 2021-11-09

**Authors:** Robert E. Kearney

**Affiliations:** 1 Department of Biomedical Engineering, McGill University, Montreal, QC, Canada

In this work the authors compare manual measures of heart rate and respiratory with measures obtained through the automated analysis of pulse oximetry and capnograph signals. The stated objective is to determine the threshold to be used in evaluating the accuracy of new continuous monitors. The major finding was that the different methods had low bias but large random errors. Given this finding it is not clear how the results of this paper contribute to its stated objectives. Indeed, the authors conclude that appropriate clinical thresholds should be selected a priori.

 I have several concerns with the paper as it stands mostly related to the methods:

1. I had difficulty understanding exactly what measures were being compared. As I understand it: For heart rate the measures were: (1) The average heart rate over a 60 second period computed as: The reciprocal of the number of heart beats manually over the period. (2) The median of the reciprocal of the beat to beat to beat interval computed by the Masimo device. For respiratory rate the two measures were (1) The reciprocal of the number of breaths in period determined by manual analysis of the capnograph and (2) The reciprocal of the median breath duration computed over the same period using a computer algorithm. This is correct, then the authors are not actually comparing measures of heart rate and respiratory rate but rather measures of their average values computed over a one-minute period. A difficulty with this is that this provides no measure of the accuracy of measures of either HR or RR variability. The authors need to make it clear exactly what they were comparing, justify their choice, and explain why they used a one-minute period.
2. The authors use confidence intervals and limits of agreement derived from the Bland Altman plots to assess the differences between measures. My understanding is that the validity of these measures depends on the assumption that the differences between measures are normally distributed. Did the authors validate this assumption?
3. The automatic analysis of the capnograph was done using an algorithm developed by the authors and described in a conference paper. There is no discussion of how this algorithm was validated or what its expected accuracy was.
4. The paper would be greatly improved by the inclusion of a figure showing a typical data record with manual markings and a clear definition of what the various measures were. 
5. One of the authors' conclusions is that a wider spread between the LOA values should be allowed to account for intra- and inter-neonate variability. It is not clear to me why this should be.

Is the work clearly and accurately presented and does it cite the current literature?

Partly

If applicable, is the statistical analysis and its interpretation appropriate?

Partly

Are all the source data underlying the results available to ensure full reproducibility?

Yes

Is the study design appropriate and is the work technically sound?

Partly

Are the conclusions drawn adequately supported by the results?

Partly

Are sufficient details of methods and analysis provided to allow replication by others?

No

Reviewer Expertise:

Biomedical signal and system analysis

I confirm that I have read this submission and believe that I have an appropriate level of expertise to state that I do not consider it to be of an acceptable scientific standard, for reasons outlined above.

# Peer review: Reviewer response for version 2

**DOI:** 10.21956/gatesopenres.14653.r31256

**Published:** 2021-11-01

**Authors:** Kevin Baker

**Affiliations:** 1 Malaria Consortium, London, UK; 2 Karolinska Institute, Stockholm, Sweden

Thanks for the opportunity to review this important work. It is a very well written piece and congratulations to the authors on describing this work so clearly. 

 I think this work builds on a number of previous studies and as there are so many groups working on RR at the moment I think it would have be useful to situate this work in relation to the previous studies and articles (listed below) and discussions that have taken place, I think by showing that these results are similar it strengthens the arguments around what the apriori thresholds should be. Again these were discussed at a UNICEF meeting in 2019 and this could be referenced also - to show that these findings match global discussions.

 In measuring RR we know that movement has a huge impact on the variability of RR - this is not well described in the piece. The authors mention "To minimize variability in our data set, we used only RR epochs that appeared to be regular based on visual inspection. Although these segments were selected based on predefined criteria, a majority (167/297) were discarded as the extreme variability seen in some recordings would have made reproducible manual counting of breaths impossible". Does this mean you removed RR epochs or instances where there was a lot of movement? While I agree that it is good to reduce variability I would be concerned that by removing the highly variable epochs the authors are not reflecting a true RR. Apologies if I misunderstood the methods here. 

 Articles to reference in the background:

- Carina *et al.* (2021 ^1^).
- Stratil *et al.* (2021 ^2^).
- Baker *et al.* (2019 ^3^).

Is the work clearly and accurately presented and does it cite the current literature?

Yes

If applicable, is the statistical analysis and its interpretation appropriate?

Yes

Are all the source data underlying the results available to ensure full reproducibility?

Yes

Is the study design appropriate and is the work technically sound?

Yes

Are the conclusions drawn adequately supported by the results?

Yes

Are sufficient details of methods and analysis provided to allow replication by others?

Yes

Reviewer Expertise:

Pneumonia malaria child health

I confirm that I have read this submission and believe that I have an appropriate level of expertise to confirm that it is of an acceptable scientific standard.

## References

[1] : Back to Basics in Paediatric Pneumonia—Defining a Breath and Setting Reference Standards to Innovate Respiratory Rate Counting. *Journal of Tropical Pediatrics* .2021;67(1) : 10.1093/tropej/fmaa112 10.1093/tropej/fmaa112 PMC794838333280051

[2] : Evaluating the Interrater Agreement and Acceptability of a New Reference Tool for Assessing Respiratory Rate in Children under Five with Cough and/or Difficulty Breathing. *Journal of Tropical Pediatrics* .2021;67(2) : 10.1093/tropej/fmab046 10.1093/tropej/fmab046 PMC820184134124753

[3] : Performance of Four Respiratory Rate Counters to Support Community Health Workers to Detect the Symptoms of Pneumonia in Children in Low Resource Settings: A Prospective, Multicentre, Hospital-Based, Single-Blinded, Comparative Trial. *EClinicalMedicine* .2019;12: 10.1016/j.eclinm.2019.05.013 20-30 10.1016/j.eclinm.2019.05.013 31388660PMC6677646

# Peer review: Reviewer response for version 2

**DOI:** 10.21956/gatesopenres.14653.r31230

**Published:** 2021-10-18

**Authors:** Gordon B. Drummond

**Affiliations:** 1 Department of Anaesthesia, Critical Care, and Pain Medicine, University of Edinburgh, Edinburgh, UK

I've now developed one further question about the data measurement process. It is this:

 If respiratory rate was measured from exactly the same epoch, and standard criteria were used for defining a breath, is it necessarily also true that exactly the same breaths were counted? I suggest this is not the case, and this is for the reason the authors imply, but do not elucidate: the monitor-derived value is calculated from "past values" and displayed in the "present". The observer based measure is then taken from events that happen from that time forward. I would suggest a simple test of the monitors, using a simulated sample of waveforms, whose duration could be abruptly changed, say from 2 seconds to 2.5 seconds, would substantially elucidate the capacity of the device to reflect the "real now" signal that the observer has been set to observe. A diagram to show the relative times of what is measured by a monitor, and an observer, relative to the "reference mark" time, would be helpful for the reader to grasp these concepts.

Is the work clearly and accurately presented and does it cite the current literature?

Partly

If applicable, is the statistical analysis and its interpretation appropriate?

Not applicable

Are all the source data underlying the results available to ensure full reproducibility?

No

Is the study design appropriate and is the work technically sound?

Partly

Are the conclusions drawn adequately supported by the results?

No

Are sufficient details of methods and analysis provided to allow replication by others?

No

Reviewer Expertise:

NA

I confirm that I have read this submission and believe that I have an appropriate level of expertise to confirm that it is of an acceptable scientific standard, however I have significant reservations, as outlined above.

# Response

**Published:** 2021-11-10

**Authors:** Jesse Coleman

Dear Dr. Drummond,

 Thank you for providing the opportunity to respond to your query on our updated version of the manuscript titled “Identification of thresholds for accuracy comparisons of heart rate and respiratory rate in neonates” at *Gates Open Access*. We appreciate your ongoing effort to review the manuscript and are grateful for your further comment. Below is our response to your query:

**Query**: *If respiratory rate was measured from exactly the same epoch, and standard criteria were used for defining a breath, is it necessarily also true that exactly the same breaths were counted? I suggest this is not the case, and this is for the reason the authors imply, but do not elucidate: the monitor-derived value is calculated from "past values" and displayed in the "present". The observer based measure is then taken from events that happen from that time forward. I would suggest a simple test of the monitors, using a simulated sample of waveforms, whose duration could be abruptly changed, say from 2 seconds to 2.5 seconds, would substantially elucidate the capacity of the device to reflect the "real now" signal that the observer has been set to observe. A diagram to show the relative times of what is measured by a monitor, and an observer, relative to the "reference mark" time, would be helpful for the reader to grasp these concepts.*

**Response**: Thank you for highlighting this tricky aspect of breath identification and respiratory rate comparison. We were able to match the exact breath. Our team had access to the raw (instantaneous) CO _2_ waveform data, recorded at approximately 20 Hz. We only analyzed the raw waveform data and counted the number of breaths. Furthermore, each 60-second epoch was isolated; no data from before or after the epoch was included in any calculation or analysis. Rather, individual breaths were counted using two different breath counting methods; 1, Study team members manual breath counting from capnograms using standardized breath identification rules, and 2, Algorithm-derived breath identification developed in MATLAB. Your suggestion about simulated respiratory rate comparisons may be an alternative if we did not have this very precise breath identification or if some method of filtering or averaging of RR was used.

# Peer review: Reviewer response for version 1

**DOI:** 10.21956/gatesopenres.14469.r30945

**Published:** 2021-08-03

**Authors:** Gordon B. Drummond

**Affiliations:** 1 Department of Anaesthesia, Critical Care, and Pain Medicine, University of Edinburgh, Edinburgh, UK

The exact hypothesis of this study is hard to discern. In their abstract and introduction, the authors imply that innovative, *non-invasive measurement* technologies that use advanced measures of vital signs such as heart rate variation and transient deceleration (citation 2) can be used to improve outcome in infants in resource-constrained settings such as low and middle income countries, but the paper then describes a comparison of nurse observation with continuous measures available from electronic monitors, with the stated aim of defining the accuracy of methods to continuously measure physiological events. Such comparisons have been done, and they cite a substantial review (citation 4).

 The introduction then ends with this statement of the study aim: “the clinical reference technology verification processes conducted to determine appropriate heart rate (HR) and respiratory rate (RR) thresholds in subsequent accuracy comparisons.”

 However the methods then state the aim is “to identify the natural variation in neonatal HR and RR in order to identify appropriate accuracy thresholds for use in an accuracy comparison of MCPM technologies.”

 So, we have at least three alternative study aims: the third I’d consider to be the most useful aim, comparing MCPM methods: unlikely to be answered when comparing clinicians with monitors, but could be answered with the data gathered.

 At this point, I felt that some sensible and more exact definitions are required, for words such as accuracy, repeatability, agreement, threshold, precision perhaps – as stated in citation 6, by two of the authors of the present paper.

 What is “Repeatability”? If we accept that the result of a 60 second counting period will differ, from one observation to the next, because the components of the measure (the duration of each breath, or the interval between photoplethysmograph pulse waves) are randomly different, then the only mechanism available to improve the *estimate* of the overall frequency is to increase the size of the sample: this is the law of large numbers, a statistical rule that has been known for several centuries in one form or another.

 Bland and Altman, when first introducing their extremely popular method, used an example of spirometry: a single measure made first with one device and then with an alternative device. It’s quite possible that two repeat FVC manoeuvres with the same device would differ: within subject variation. This is a more substantial problem in this study, as the authors state:

 “Furthermore, a ±2 breaths per minute or 5% spread in LOA is smaller than random and natural within-neonate physiologic variability (11.5% in this study [unpublished data]) and would result in unrealistically stringent thresholds”. The degree of within subject variation is evident also from Figure 3. The phenomenon was noted by Simoes *et al.* (1991 ^1^).

 So we have a small number of intrinsically variable events. So, for a fair comparison of two methods, a necessary requirement is to ensure that the events being measured are the same, exactly the same sample has been taken. If the pulse-wave derived rate from the machine is of a different series of waves (i.e the time period is not EXACTLY the same) than those counted by the nurse, they are already going to be affected by within subject variation as well as the variation between the methods. The methods state: “Manual measures were every 10 minutes for the first hour and once per hour of participation thereafter: were the manual and monitor measures exactly timed to coincide? And, was there any time trend in the patients studied for longer times?

 Of course, Bland and Altman had to subsequently refine their method, to separately account for repeated measures in multiple subjects, and at the same time they introduced the concept of confidence intervals for the limits of agreement. Looking at figure 3, there’s a lot of variation: it would be helpful to plot the CI for the LOA on the Bland and Altman plots.

 However, I would suggest that the most useful thing to do would be to carefully analyse repeated random samples from the electronic records, looking at precise time intervals, so that the intrinsic variation could be quantified, and study how different sample sizes might affect reliability of the rate values. We have done this for respiratory rate in acutely ill adults (Drummond *et al.*, 2020 ^2^).

 Using 30 second periods of observation gave an interquartile range of respiratory rates of 3.4 breath/minute, whereas samples taken for 120 seconds had an IQR of 2.5. Using the techniques the authors describe here, why not sample for 5 minutes?

 Availability of these records would be very useful to other workers! More analysis of the monitor records is also important since it appears that rate is not, in itself, perhaps the most important signal. For example, others have found that short-term heart and respiratory rate variability make a significant contribution to illness scoring systems (Saria *et al.*, 2010 ^3^).

**Small points:**

- Abstract: “Automated measurement technologies typically use median values”. I’m afraid that my experience is that manufacturers of monitors rarely tell what they use: some sort of exponential averaging or filtering seems more likely. It would be good to have this statement substantiated (if possible).
- Abbreviations make reading difficult. “multiparameter continuous physiological monitoring (MCPM) technologies” is subsequently used as MCPM technologies (17 characters) throughout the paper. Why not just use “continuous monitors” (19 characters)?
- Pulse plethysmography may not be an accurate measure of heart rate variability. ECG monitoring might be better. I realise that ECG has its drawbacks!

Is the work clearly and accurately presented and does it cite the current literature?

Partly

If applicable, is the statistical analysis and its interpretation appropriate?

Not applicable

Are all the source data underlying the results available to ensure full reproducibility?

No

Is the study design appropriate and is the work technically sound?

Partly

Are the conclusions drawn adequately supported by the results?

No

Are sufficient details of methods and analysis provided to allow replication by others?

No

Reviewer Expertise:

Expertise in respiratory monitoring and data analysis

I confirm that I have read this submission and believe that I have an appropriate level of expertise to state that I do not consider it to be of an acceptable scientific standard, for reasons outlined above.

## References

[1] : Respiratory rate: measurement of variability over time and accuracy at different counting periods. *Arch Dis Child* .1991;66(10) : 10.1136/adc.66.10.1199 1199-203 10.1136/adc.66.10.1199 1953002PMC1793530

[2] : Current clinical methods of measurement of respiratory rate give imprecise values. *ERJ Open Res* .2020;6(3) : 10.1183/23120541.00023-2020 10.1183/23120541.00023-2020 33015146PMC7520170

[3] : Integration of early physiological responses predicts later illness severity in preterm infants. *Sci Transl Med* .2010;2(48) : 10.1126/scitranslmed.3001304 48ra65 10.1126/scitranslmed.3001304 20826840PMC3564961

# Response

**Published:** 2021-09-27

**Authors:** Jesse Coleman

We thank Dr. Drummond for their time and effort in providing valuable feedback and we are grateful to you for the insightful comments. We have been able to incorporate changes to reflect most of the suggestions you provided. Our responses to the points raised are below:

**Point 1**: *The exact hypothesis of this study is hard to discern. In their abstract and introduction, the authors imply that innovative, non-invasive measurement technologies that use advanced measures of vital signs such as heart rate variation and transient deceleration (citation 2) can be used to improve outcome in infants in resource-constrained settings such as low and middle income countries, but the paper then describes a comparison of nurse observation with continuous measures available from electronic monitors, with the stated aim of defining the accuracy of methods to continuously measure physiological events. Such comparisons have been done, and they cite a substantial review (citation 4).*

*The introduction then ends with this statement of the study aim: “the clinical reference technology verification processes conducted to determine appropriate heart rate (HR) and respiratory rate (RR) thresholds in subsequent accuracy comparisons.”*

*However the methods then state the aim is “to identify the natural variation in neonatal HR and RR in order to identify appropriate accuracy thresholds for use in an accuracy comparison of MCPM technologies.”*

*So, we have at least three alternative study aims: the third I’d consider to be the most useful aim, comparing MCPM methods: unlikely to be answered when comparing clinicians with monitors, but could be answered with the data gathered.*

**Response**: Thank you for identifying that the language used to introduce the topic might not align with the stated goals of the manuscript. The reviewer is correct that the 3rd aim is what we have addressed. We will be modifying the language in the introduction section to read “ **This study describes the verification processes we conducted with a clinical reference technology in order to determine appropriate heart rate (HR) and respiratory rate (RR) accuracy thresholds to use in subsequent new patient monitoring technology accuracy comparisons.**”

**Point 2**: *At this point, I felt that some sensible and more exact definitions are required, for words such as accuracy, repeatability, agreement, threshold, precision perhaps – as stated in citation 6, by two of the authors of the present paper*.

**Response**: We agree with your recommendation to clarify the definitions of some keywords. We will be adding definitions for the suggested terms to the definitions table to read as follows:

**Accuracy: The closeness a measured value is from the true value**

**Repeatability: The closeness of the results of successive measurements of the same measure**

**Agreement (between measures): The consistency between two sets of measurements**

**Accuracy Threshold: A pre-specified value used to determine if a set of measurements has achieved a sufficient accuracy when compared with a reference value**

**Precision: The closeness of measurements to each other**

**Point 3**: *What is “Repeatability”? If we accept that the result of a 60 second counting period will differ, from one observation to the next, because the components of the measure (the duration of each breath, or the interval between photoplethysmograph pulse waves) are randomly different, then the only mechanism available to improve the estimate of the overall frequency is to increase the size of the sample: this is the law of large numbers, a statistical rule that has been known for several centuries in one form or another.*

*Bland and Altman, when first introducing their extremely popular method, used an example of spirometry: a single measure made first with one device and then with an alternative device. It’s quite possible that two repeat FVC manoeuvres with the same device would differ: within subject variation. This is a more substantial problem in this study, as the authors state: “Furthermore, a ±2 breaths per minute or 5% spread in LOA is smaller than random and natural within-neonate physiologic variability (11.5% in this study [unpublished data]) and would result in unrealistically stringent thresholds”. The degree of within subject variation is evident also from Figure 3. The phenomenon was noted by Simoes et al. (1991 1).*

*So we have a small number of intrinsically variable events. So, for a fair comparison of two methods, a necessary requirement is to ensure that the events being measured are the same, exactly the same sample has been taken. If the pulse-wave derived rate from the machine is of a different series of waves (i.e the time period is not EXACTLY the same) than those counted by the nurse, they are already going to be affected by within subject variation as well as the variation between the methods. The methods state: “Manual measures were every 10 minutes for the first hour and once per hour of participation thereafter: were the manual and monitor measures exactly timed to coincide?*

**Response**: You have raised a critically important issue of synchronization. We apologize that we did not emphasize the importance in the original draft. We have updated our methods section to clearly describe the synchronization methods we used to ensure that all data was precisely temporally aligned. The new wording will read as follows: “ **To ensure temporal alignment between measurements, HR and RR epochs were synchronized across source data devices. For HR, alignment was done using a timestamp in REDCap that was set by the study nurse as HR counting was initiated. Before analysis, this timestamp was synchronized with the same timestamp in the custom Android application. Both the REDCap and Android servers were connected via the internet to a Network Time Protocol (NTP) server. Alignment of RR epochs was based on the Android application timestamp. All RR waveforms were compared visually to further ensure epoch synchronization.**” With the definition and clarification made, we feel that testing the repeatability and agreement of the two methods is reasonable. 

**Point 4**: *And, was there any time trend in the patients studied for longer times?*

**Response: **Thank you for asking this question. We do have respiratory rate data on patients studied for longer times that has been submitted and is currently under review elsewhere.

**Point 5**: *Of course, Bland and Altman had to subsequently refine their method, to separately account for repeated measures in multiple subjects, and at the same time they introduced the concept of confidence intervals for the limits of agreement. Looking at figure 3, there’s a lot of variation: it would be helpful to plot the CI for the LOA on the Bland and Altman plots.*

**Response**: Thank you for the helpful suggestion. We will be updating the Bland and Altman plots to include the CI for the LOA throughout.

**Point 6**: *However, I would suggest that the most useful thing to do would be to carefully analyse repeated random samples from the electronic records, looking at precise time intervals, so that the intrinsic variation could be quantified, and study how different sample sizes might affect reliability of the rate values. We have done this for respiratory rate in acutely ill adults (Drummond et al., 2020).*

*Using 30 second periods of observation gave an interquartile range of respiratory rates of 3.4 breath/minute, whereas samples taken for 120 seconds had an IQR of 2.5. Using the techniques the authors describe here, why not sample for 5 minutes?*

**Response**: Thank you for this suggestion. It would have been important to look at repeated random samples if we did not have temporally aligned data. However, in the case of our study, it is unnecessary because we can compare measurements from the same epoch. We do have a manuscript in preparation that will address this clinical issue of counting. Contrary to your suggestion we found the agreement deteriorated in observations over one minute. In this manuscript, we focus on the thresholds we would consider appropriate for a subsequent method comparison study.

**Point 7**: *Availability of these records would be very useful to other workers ! More analysis of the monitor records is also important since it appears that rate is not, in itself, perhaps the most important signal. For example, others have found that short-term heart and respiratory rate variability make a significant contribution to illness scoring systems (Saria et al., 2010).*

**Response**: Thank you for this suggestion. We completely agree and one of the reasons for the current research is to facilitate the introduction of high-quality vital sign monitors in resource-constrained settings. Our team works closely with manufacturers, who we will recommend include notifications of short-term HR and RR variability. Also, All the data will be made available for other researchers to allow them to address these important considerations.

Small points

**Small point 1**: *Abstract: “Automated measurement technologies typically use median values”. I’m afraid that my experience is that manufacturers of monitors rarely tell what they use: some sort of exponential averaging or filtering seems more likely. It would be good to have this statement substantiated (if possible).*

**Response**: You are correct. A median is a simple filter and other filters may also be used and would be considered a trade secret. I think the key point here is that a count of breaths is likely to be very different from any filter method that might be used. We will be adjusting the text to read  “ **typically use a smoothing or averaging filter**”

**Small point 2**: *Abbreviations make reading difficult. “multiparameter continuous physiological monitoring (MCPM) technologies” is subsequently used as MCPM technologies (17 characters) throughout the paper. Why not just use “continuous monitors” (19 characters)?*

**Response**: Thank you for this suggestion. We understand that acronyms can be difficult to read. To facilitate readability we will integrate your suggested terminology to read “ **continuous monitors**” throughout.

**Small point 3**: *Pulse plethysmography may not be an accurate measure of heart rate variability. ECG monitoring might be better. I realise that ECG has its drawbacks!*

**Response**: We would agree that there is a difference between heart variability derived from ECG and that derived from pulse plethysmography. We have previously compared the measures of heart rate variability between ECG and plethysmography in children. This agreement is dependent on an appropriate sampling rate of the plethysmogram. Noting your comment, we will add the following text to the discussion: “ **Pulse plethysmography may not be an accurate measure of HR variability due to innate technology limitations. Future studies looking at HR variability should consider using ECG monitoring, despite having its own limitations. ^1^**”

1. Reference: Dehkordi, P., Garde, A., Karlen, W., Wensley, D., Ansermino, J. M., & Dumont, G. A. (2013). Pulse rate variability compared with Heart Rate Variability in children with and without sleep disordered breathing. 2013 35th Annual International Conference of the IEEE Engineering in Medicine and Biology Society (EMBC), 2013, 6563–6566. https://doi.org/10.1109/EMBC.2013.6611059
